# Mortality affects adaptive allocation to growth and reproduction: field evidence from a guild of body snatchers

**DOI:** 10.1186/1471-2148-10-136

**Published:** 2010-05-07

**Authors:** Ryan F Hechinger

**Affiliations:** 1 Marine Science Institute and the Department of Ecology, Evolution & Marine Biology University of California Santa Barbara, CA 93106-6150, USA

## Abstract

**Background:**

The probability of being killed by external factors (extrinsic mortality) should influence how individuals allocate limited resources to the competing processes of growth and reproduction. Increased extrinsic mortality should select for decreased allocation to growth and for increased reproductive effort. This study presents perhaps the first clear cross-species test of this hypothesis, capitalizing on the unique properties offered by a diverse guild of parasitic castrators (body snatchers). I quantify growth, reproductive effort, and expected extrinsic mortality for several species that, despite being different species, use the same species' phenotype for growth and survival. These are eight trematode parasitic castrators—the individuals of which infect and take over the bodies of the same host species—and their uninfected host, the California horn snail.

**Results:**

As predicted, across species, growth decreased with increased extrinsic mortality, while reproductive effort increased with increased extrinsic mortality. The trematode parasitic castrator species (operating stolen host bodies) that were more likely to be killed by dominant species allocated less to growth and relatively more to current reproduction than did species with greater life expectancies. Both genders of uninfected snails fit into the patterns observed for the parasitic castrator species, allocating as much to growth and to current reproduction as expected given their probability of reproductive death (castration by trematode parasites). Additionally, species differences appeared to represent species-specific adaptations, not general plastic responses to local mortality risk.

**Conclusions:**

Broadly, this research illustrates that parasitic castrator guilds can allow unique comparative tests discerning the forces promoting adaptive evolution. The specific findings of this study support the hypothesis that extrinsic mortality influences species differences in growth and reproduction.

## Background

How much should any organism grow? How much should it reproduce? These fundamental questions are tightly related. Increased growth brings larger size. Larger size typically brings greater reproductive rates and survivorship [1]. Thus, investment in growth represents investment in future reproduction [2,3]. However, investment in growth theoretically diverts resources from current reproduction, resulting in an allocation trade-off. Theory indicates that this trade-off is strongly influenced by extrinsic mortality (the probability of being killed by external factors) [e.g., [4-9]]. Here, I perform what I believe to be the first clear test of whether extrinsic mortality drives among-species differences in allocation to growth and reproduction. This was possible by using a study system recently identified [10] as being particularly suitable for comparative examination of adaptation: several parasitic castrator species—the individuals of which infect and take over the bodies of the same host species—and their uninfected host.

Extrinsic mortality should affect the trade-off between growth and reproduction because increased mortality means a smaller chance of surviving long enough to experience the reproductive gains of having grown to a larger size [2]. Thus, greater adult mortality should select for less growth and a greater reproductive effort (proportional allocation to current reproduction). Conversely, lower mortality should select for more growth and a lower reproductive effort. This theory applies to species that continue to grow after maturation (indeterminant growers). It also applies to those that cease growth after maturation (determinant growers), with the emphasis being on how mortality influences growth before maturation. The few clear empirical examinations of whether extrinsic mortality influences allocation to growth and reproduction in nature have been cross-population intraspecific studies [e.g., see particularly [11,12] and related work, see also [13,14]]. There is little or no data available to allow a clear cross-species examination of this hypothesis, at least partly due to the difficulties of acquiring the necessary information on extrinsic mortality.

Parasitic castrators may facilitate investigation of such difficult to test questions. An important feature of parasitic castrators is that a single castrator takes over a host's body and then uses that body for the castrator's sole benefit. A parasitic castrator infection typically completely and permanently blocks host reproduction, replacing host reproductive tissues with parasite tissues [15-19]. Thus, the castrated host is dead concerning its fitness, its reproductive value reduced to zero [15,16,18,20-23]. However, the castrator can potentially operate the stolen host body for years [15,16,24]. Further, parasitic castrators manipulate the physiology of stolen host bodies, for instance by secreting hormones and by modulating gene expression (e.g., [25] and see reviews in [15,16]). These factors—particularly the castrated host's zero residual reproductive value in conjunction with the castrator's manipulation of the stolen host physiology—lead to the realization that selection will operate on parasitic castrators, not on reproductively dead hosts, regarding adaptive resource allocation of the stolen host bodies [10,16,20,23].

Situations where multiple parasitic castrator species specialize on the same host species should enable powerful analyses concerning optimal allocation schemes [10]. Such diverse parasitic castrator guilds provide a group of species lacking many confounds that can characterize both intraspecific and interspecific comparative studies. Each species lives in the same environment, at the same time. Further removing confounds, each species uses the same physiological machinery (the stolen host species' body) for growth and survival. Another advantage is that castrator-infected and uninfected hosts can compete for the same resources [e.g., [26]]. Thus, the direct effect of parasitic castrator-driven mortality on optimal allocation strategies may not be confounded by the indirect effect of increasing resources, as can occur when predator-driven mortality reduces prey population density and frees up resources [e.g., [27,28]]. Importantly, despite being similar in many essential ways, the parasitic castrator species can differ from each other and from uninfected hosts regarding factors influencing their reproductive expectations [10]. Therefore, selection may generate allocation schemes that vary not only between infected and uninfected hosts, but also among different castrator species parasitizing the same host species. Such interspecific variation would provide material very useful for comparative analysis.

A parasitic castrator-host system with many species that differ in important and quantifiable factors influencing extrinsic mortality is the California horn snail, *Cerithidea californica*, and its suite of parasitic castrator trematodes. Shortly after infection, a trematode reproductively kills the snail by castrating it. The trematode stages (parthenitae) clonally replicate and shortly begin producing free-swimming offspring (cercariae), for many seasons, over many years [29,30]. Not only do trematodes reproductively kill hosts, but the trematode species also kill each other [29,30]. Dominant trematode species kill subordinate species when they both infect the same horn snail [29,30]. Interspecific dominance occurs in a predictable, hierarchical way, and is largely determined via interactions between the individual, clonal parthenitae that compose a single species' infection mass [29-32]. The trematode species vary in the level of extrinsic mortality caused by dominant species [29,30,33]. The degree of this extrinsic mortality is based upon the likelihood of encountering a dominant species, which is jointly determined by a species' dominance rank and its spatial distribution within estuaries [33,34]. For instance, a subordinate species could experience low mortality if it tends to occur in areas lacking dominant species. Despite varying extrinsic mortality risk from dominant trematodes, the background extrinsic mortality of the different infected and uninfected snails (e.g., due to predation and abiotic stress) appears to be relatively constant [35,36]. Because we can estimate the "differential mortality" caused by castration or death by dominant trematodes [33], this group of species provides a system very amenable to studying how extrinsic mortality influences optimal allocation to growth and reproduction.

Here, I present data and analyses examining how extrinsic mortality influences allocation to growth and reproduction for eight trematode parasitic castrator species and their uninfected host, the California horn snail. The backbone of this study is growth and infection information from 1,686 snails, recaptured from a wide range of field growing conditions. I estimated reproductive effort for these trematode species using published information on parasite/host tissue ratios (equivalent to a gonadosomatic index) combined with the new growth data. I calculated differential extrinsic mortality (proportion of recruits killed by other trematode species) using information on the trematode species' dominance hierarchy and infection prevalences at the study sites. Recognizing that a trematode takes over the host body, I generally refer to snails castrated by a trematode species simply as that trematode species. As predicted, across species, growth allocation decreased with increasing mortality, while reproductive effort increased. Further, most parasitic castrator species grew more than did uninfected hosts—reflecting their average longer life expectancies. Thus, these findings support a fundamental element of life history theory, which itself provides a simple explanation for cases of "gigantism" induced by parasitic castrator infection.

## Results

### Variation in growth

**Figure 1:**
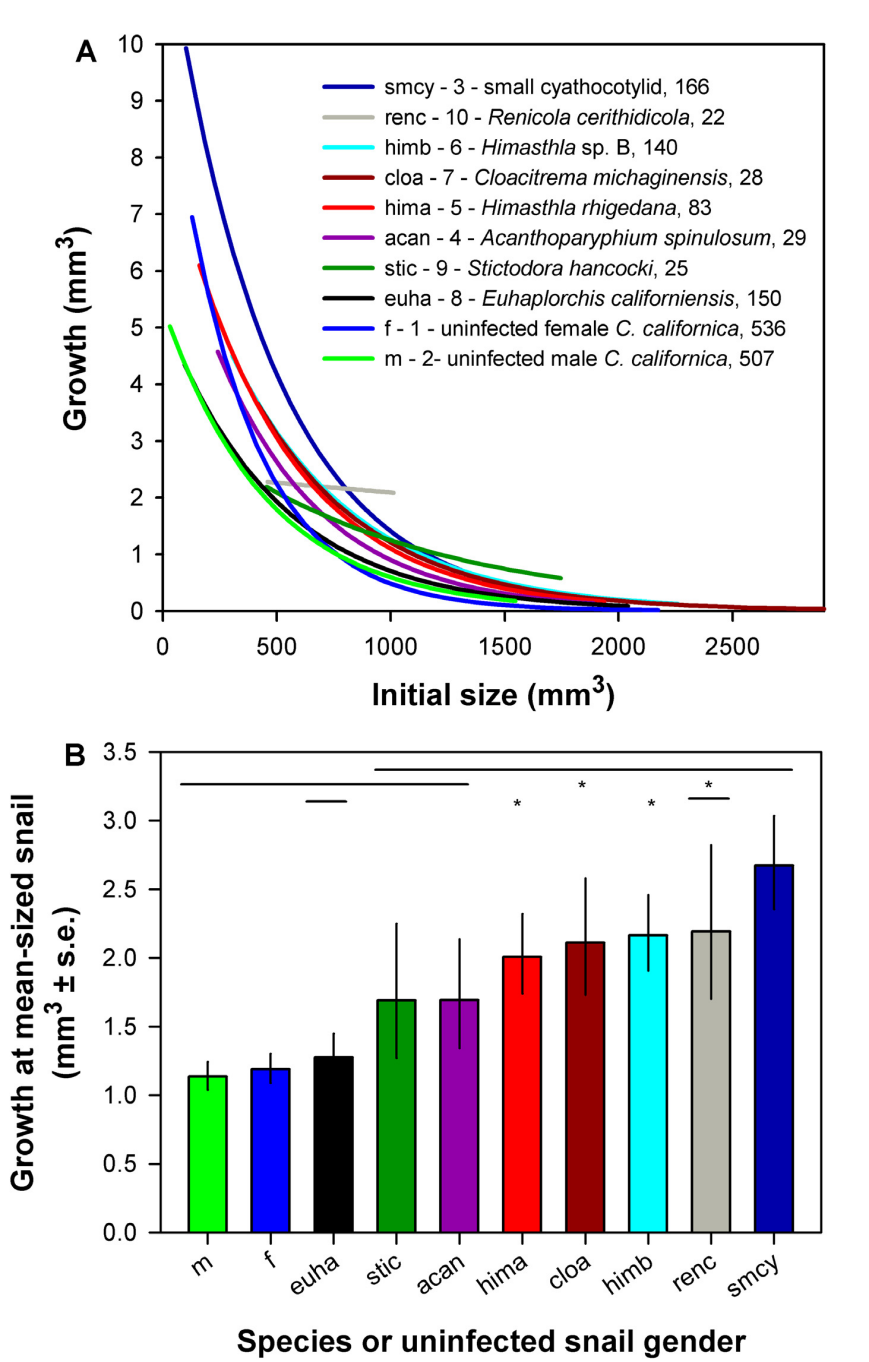
**Variation in growth for eight different species of trematode parasitic castrators (operating stolen host bodies) and for both genders of uninfected horn snails**. (A) Curves denote mean size-specific growth rates over the size-range sampled for each species (as estimated from a generalized linear model, Table 1). Species in legend are in the descending order of the growth curves as encountered at the mean-sized snail (705 mm^3^, or ~ 25.9 mm length), and sample sizes directly follow the species' names (Additional file 1, Table S1 lists the species' taxonomic families). (B) Variation in growth at the mean-sized snail. Results of pair-wise comparisons indicted. Bars sharing an equal-height line are not significantly different at the nominal *P *= 0.05 level. Asterisks denote additional non-significant differences when controlling for multiple comparisons by holding the False Discovery Rate to 0.05. Data in (A) and (B) represent 1,686 individuals spread throughout 17 sites in three southern California estuaries. Data standardized to reflect 3.5 months of growth in Carpinteria Salt Marsh channels. Other figures and the text use the species' letter and number codes.

Growth rates varied among snails infected by different trematode species and uninfected snails (Figure 1, Table 1). The two most extensively sampled trematode species (growing stolen host bodies) were also the fastest and slowest growing trematodes. The fastest growing trematode (smcy) grew up to three times faster than uninfected snails, while the slowest growing trematode (euha) grew about the same as uninfected male snails. Overall, most trematode species grew faster than uninfected snails. This appears not to be an artifact of trematodes disproportionately infecting faster-growing snails (see Additional file 1, Figure S5). The effects of species on growth rates appeared to be consistent among wetlands (Additional file 1).

**Table 1:** Statistics from the generalized linear model^a ^using observed individual growth (mm^3^) as the response variable.

Explanatory variable or statistic	df	Χ^2^	*P*
"species"^b^	9	68.68	<0.0001
Initial size	1	60.45	<0.0001
"species" × initial size	9	18.87	0.026
Estuary	2	3.79	0.15
habitat [estuary]	1	9.55	0.002
site [estuary, habitat]	13	193.68	<0.0001
growing time [estuary]	2	84.42	<0.0001
whole model	37	1046.78	<0.0001
goodness of fit (deviance)	1648	6422.59	<0.0001

^a^The model used a log-link, a Poisson error distribution, and an overdispersion parameter of 4.53. Brackets indicate nested effects.

^b^"Species" indicates species of trematode parasitic castrator and male or female uninfected snail hosts.

Growth also varied with size and, for small, uninfected snails, with gender. For all species, the smallest individuals grew fastest (Figure 1; Additional file 1, Figure S2). Consistent with previous work [37], uninfected females grew faster than uninfected males at small sizes, after which they rapidly converged with males (Figures 1; Additional file 1, Figure S3). Trematodes increased growth of infected small males far more than they did females (Additional file 1, Figure S3). Thus, gender differences in growth were generally not detectable for infected snails (Additional file 1, Figure S3). The effects of gender on growth rates also appeared to be consistent among wetlands (Additional file 1).

### Variation in extrinsic mortality

**Figure 2:**
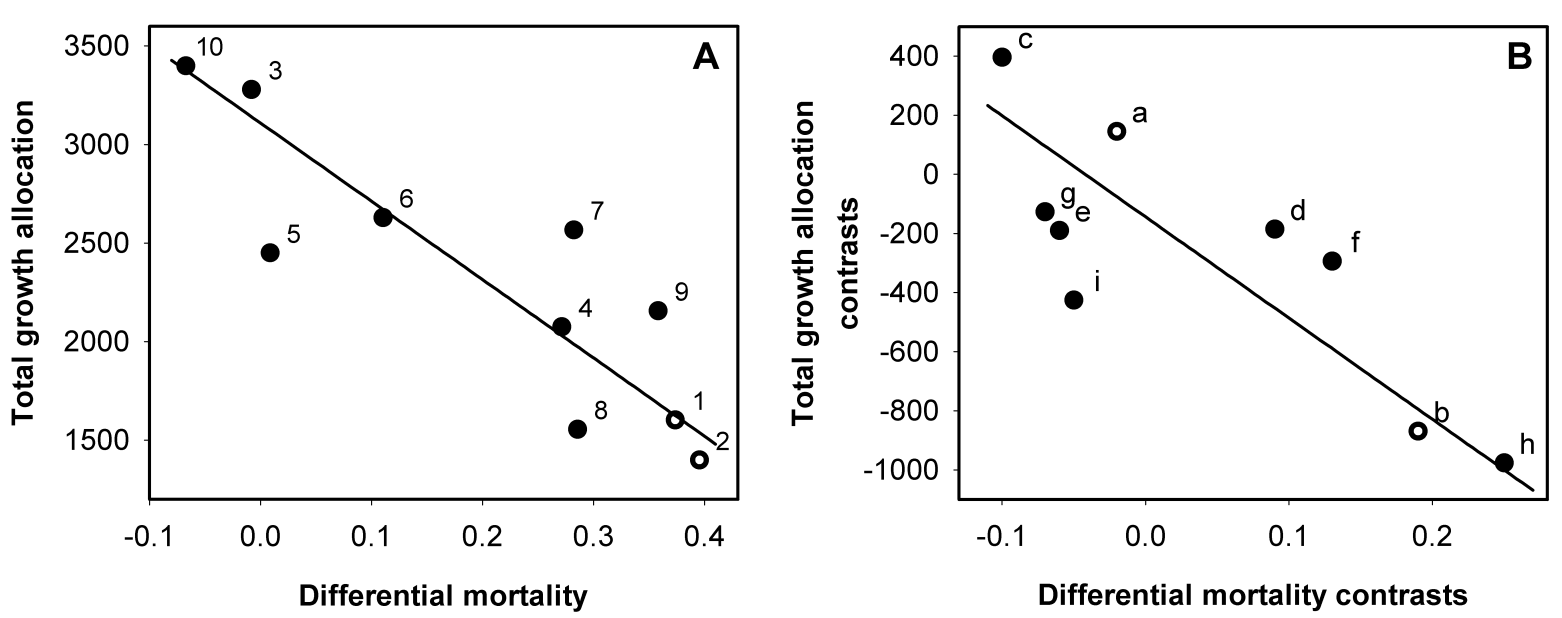
**Allocation to growth versus differential extrinsic mortality for the eight parasitic castrator trematode species (operating stolen host bodies) and both genders of uninfected horn snails**. (**A**) The analysis using species as independent data points (*r *= -0.86, *P *= 0.0014, *n *= 10). (**B) **The analysis using phylogenetically independent comparisons (*r *= -0.81, *P *= 0.0060, *n *= 9; regression through the origin: *t*_8 _= 4.0, *P *= 0.004). Total growth allocation is the area underneath the species' growth curves in Figure 1, calculated as the definite integral with limits at the minimum- and maximum-sized snail averaged across species. Differential mortality is the mean proportion of individuals killed by dominant trematodes, or, for uninfected snails, reproductively killed by trematodes. Symbol numbers indicate species as numbered in Figure 1 and Additional file 1, Table S1. Symbol letters indicate locations of contrasts in the phylogeny depicted in Figure 4. Open symbols represent uninfected snails or contrasts involving uninfected snails. Fit lines are the standard major axes, reflecting the bivariate nature of the data.

**Figure 3:**
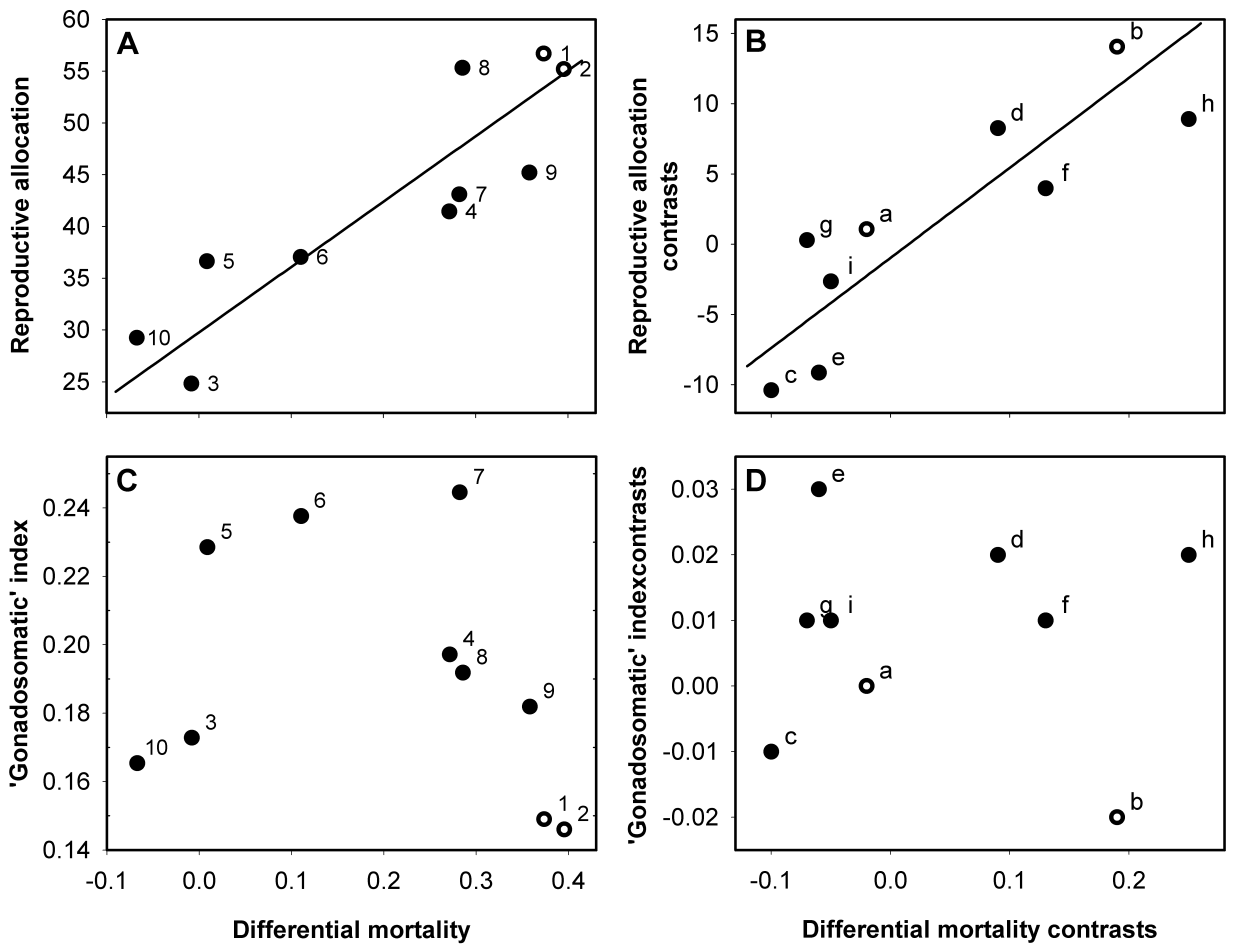
**Reproductive effort versus differential extrinsic mortality for the eight parasitic castrator trematode species (operating stolen host bodies) and both genders of uninfected horn snails**. Relative reproductive allocation increases with mortality using (**A**) raw data (*r *= 0.88, *P *= 0.0008, *n *= 10) and when using (**B**) phylogenetically independent contrasts (*r *= 0.87, *P *= 0.0026, *n *= 9; regression through the origin: *t*_8 _= 5.0, *P *= 0.0011). Relative reproductive allocation here is a modified gonadosomatic index that accounts for variation in allocation to growth by using total mass growth increment in the denominator instead of total mass. (**C**) and (**D**) show that there is no association when the raw gonadosomatic index is used to represent reproductive effort in parallel analyses (*r *= -0.24, *P *= 0.49, *n *= 10 and *r *= -0.03, *P *= 0.942, *n *= 9; regression through the origin: *t*_8 _= 0.34, *P *= 0.74). A two-stage randomization test ensured that these positive relationships were not statistical artifacts driven by the strong negative correlation of growth with mortality (see Additional file 1). Lines, symbols, and differential mortality are as in Figure 2.

The differential mortality also varied across trematode species and uninfected snails, ranging from -6.7% to 39.6% individuals lost to death by dominant trematodes, or lost to castration for uninfected snails (Figures 2, 3). The small negative mortality values represent potential facilitation, when species occur in mixed-species infections more often than expected by chance [33]. Uninfected males appeared slightly more susceptible to trematode infection than were females (Figures 2, 3; logistic regression on entire dataset, controlling for site, growing time, and size; Χ^2 ^= 4.40, df = 1, *P *= 0.0359, *n *= 1,840).

### Growth versus extrinsic mortality

Does the quantified differential mortality explain the observed differences in species' growth? As predicted, total growth allocation decreased with increasing differential mortality (Figure 2A, B). This negative correlation occurred across trematode species and both genders of uninfected snails (Figure 2A; *r *= -0.86, *P *= 0.0014, *n *= 10). An analysis using phylogenetically independent contrasts also found a negative association, confirming that taxonomic confounds did not drive the negative relationship between growth and mortality (Figure 2B; *r *= -0.81, *P *= 0.0060, *n *= 9). Uninfected snails fit into the pattern observed across trematode species (Figure 2A, B). However, the negative relationship between growth allocation and mortality did not depend on the inclusion of uninfected snails. The result was also strong and significant after excluding uninfected males and females from the analysis using species as data points (*r *= -0.80, *P *= 0.018, *n *= 8), and from the analysis using phylogenetically independent contrasts (*r *= -0.77, *P *= 0.030, *n *= 7). The case was similar upon exclusion of the two trematode species, 'stic' and 'renc', least adequately sampled at smaller sizes (species as data points: *r *= -0.84, *P *= 0.010, *n *= 8; independent contrasts: *r *= -0.78, *P *= 0.040, *n *= 7; regression through the origin: *t*_6 _= 3.1, *P *= 0.021).

Trematodes and uninfected snails did not appear to respond plastically to local differential mortality risk. There was no evidence that mean individual growth systematically varied across sites with mortality risk for any of the trematodes or both sexes of uninfected snails (Additional file 1, Figure S6; general linear model: *F*_10,82 _= 0.89, *P *= 0.54). This does not rule out the existence of any plastic responses, but that any possible responses are not responsible for the documented association between species-level differences in growth rates and differential mortality.

### Reproductive effort & gonadosomatic indices versus extrinsic mortality

Opposite to growth, relative reproductive allocation strongly increased with increasing differential extrinsic mortality (Figure 3A, B). This was true using the eight parasitic castrators and both sexes of uninfected hosts as independent data (Figure 3A; *r *= 0.88, *P *= 0.0008, *n *= 10), and when using phylogenetically independent contrasts (Figure 3B; *r *= 0.87, *P *= 0.0026, *n *= 9). Importantly, the two-step randomization test ensured that this relationship was not a spurious artifact driven by the observed cross-species growth rate variation (Additional file 1). As for the relationship between growth and mortality, uninfected snails also fit into the pattern describing the trematode species concerning the relationship between reproductive allocation and mortality. Here too, these results were robust to the exclusion of uninfected snails. The correlation was still strongly significantly positive when I excluded uninfected snails from the analysis using species as data points (*r *= 0.84, *P *= 0.0083, *n *= 8) or phylogenetically independent contrasts (*r *= 0.85, *P *= 0.019, *n *= 7). The positive association was also clear when excluding the two trematode species least adequately sampled at small sizes, 'renc' and 'stic' (species as data points: *r *= 0.90, *P *= 0.0035, *n *= 8; independent contrasts: *r *= 0.85, *P *= 0.014, *n *= 7; regression through the origin: *t*_6 _= 4.0, *P *= 0.0074).

The gonadosomatic indices for these species did not correlate with relative reproductive allocation (*r *= -0.28, *P *= 0.43, *n *= 10), or with differential mortality (Figure 3C, D).

## Discussion

This study provides perhaps the first direct empirical evidence that varying levels of extrinsic mortality across species drive adaptive interspecific differences in allocation to growth and reproduction. To my knowledge, all previous, substantial examinations of cross-species relationships between mortality and growth and reproduction [e.g., [38-41]] were unable to discern whether mortality was extrinsic or intrinsic. Therefore, it is impossible to determine directionality in previous studies. Mortality may have influenced optimal allocation, or it could have been the outcome of optimal allocation (for instance, if increased reproductive effort lowered survivorship). The current study examined allocation for several species, with estimates of a clear, extrinsic source of mortality. The trematode parasitic castrator species (operating stolen host bodies) whose individuals were more likely to die from dominant species allocated less to growth and more to reproduction than did species with greater life expectancies. Interestingly, both genders of uninfected snails fit into the patterns observed among the parasitic castrator species, allocating as much to growth and to reproduction as expected given their probability of reproductive death (castration by trematode parasites). Because the data further indicate that the species did not plastically respond to local levels of risk, the consistent species differences in allocation patterns appear to result from adaptation to different selective regimes—specifically, different overall levels of extrinsic mortality.

The findings also buttress the perspective outlined in the introduction regarding how to consider adaptation of parasitically castrated and uninfected hosts. Except for seemingly rare systems where parasitic castrator infections die with appreciable frequency and the host recuperates [e.g., [42,43]], a castrated host's reproductive value is zero. Therefore, with respect to fitness of castrated host bodies, selection will not act on host populations, but on the parasitic castrator populations (barring limited circumstances when kin selection might operate). Hence, parasitic castrators are truly 'body snatchers'. To understand uninfected host resource allocation, we apply basic life history principles to uninfected hosts. To understand resource allocation of parasitically castrated hosts, we apply the same principles to the castrators, not to their reproductively dead hosts. Supporting this point of view, in this study, castrated hosts allocated resources in the direction predicted by applying theory to the parasitic castrators, not the hosts. This perspective may help clarify future empirical and theoretical work addressing parasitic castration.

A general methodological implication of this study comes from the surprising finding that the species' gonadosomatic indices did not correlate with their relative reproductive allocation indices. The relative reproductive allocation index directly factored in variation in allocation to growth. It should therefore more accurately indicate reproductive effort than should the completely static gonadosomatic index. Indeed, the relationship between extrinsic mortality and reproductive effort was only apparent when using the relative reproductive allocation index. The apparent inadequacy of the gonadosomatic index to compare reproductive efforts of these species may partly explain the inconsistent relationship between that index and dominance rank documented in Hechinger et al. [10]. More broadly, the incongruence between the two indices suggests researchers must take extra caution when using the gonadosomatic index in comparative estimates of reproductive effort for organisms with indeterminate growth, or any species where allocation to somatic tissues may strongly vary.

An interesting issue arises regarding possible differences in overall productivity between uninfected and infected snails. Despite having a high relative reproductive allocation (driven by low growth rates), it is striking that uninfected snails had among the lowest gonadosomatic indices and the lowest growth rates. Thus, compared to the average trematode species operating stolen host bodies, uninfected snails may allocate *absolutely *less to both direct reproductive output and to growth. If so, this may reflect advantages of trematode clonal reproduction compared to the sexual dioecy of uninfected snails. Future work should detail the entire energy budgets (including behavioral expenditures) for parasitic castrator species and their uninfected hosts to more fully understand absolute and relative species differences in resource allocation. Such work should also factor in offspring survivorship, for which there is coarse evidence that it influences optimal allocation schemes of trematode parasitic castrators [10,44].

The growth findings here also directly bear on an issue that has long interested students of parasitic castrator systems. For decades, researchers have noted that parasitic castrators—particularly trematodes in snails—can cause gigantism, increasing growth of infected hosts relative to uninfected hosts [reviewed in [16-18,37]]. However, gigantism was not detected in the first three studies that directly quantified growth for long-lived marine snails infected and uninfected by trematode parasitic castrators [26,37,45]. This lack of detecting gigantism led to the development of conceptual theory explaining why gigantism should not occur in long-lived host species (those living > 1.5 years [37] or 4 years [46]). This theory relied on postulated differences in the allocation schemes of host species with different longevities and on parasitic castrator constraints on energy use. However, the results presented here, along with those of Miura et al. [47] on a single trematode species, clearly indicate that trematode parasitic castrators can cause gigantism in long-lived marine snails. Some workers have postulated that gigantism might be adaptive for the parasitic castrators, by increasing fecundity or survivorship [e.g., [15,16,18,23,47,48]]. However, it appears there has never been an explanation for why uninfected hosts would not also benefit from growing larger. The perspective and findings of this paper provide a simple potential answer: uninfected hosts would not benefit from growing larger because they will not live as long. Higher extrinsic mortality for uninfected hosts compared to parasitic castrators can select for a lower allocation to growth for uninfected hosts compared to the amount allocated by parasitic castrators. Minchella [49] did predict that gigantism would tend to occur for longer-lived host species, but for a different reason. He hypothesized gigantism was adaptive for the host, by increasing the probability of outliving the castrator infection. This seems unlikely to provide a general explanation, given that long-lived hosts do not generally outlive infection [16,29,30,50,51]. If gigantism is comparatively frequent in longer-lived host species, it may be because the longevity of such hosts makes more possible the differential life expectancies necessary to select for detectably different allocation schemes between parasitic castrators and uninfected hosts.

The finding that trematodes generally increased growth appears to contradict previous work on the same system. Sousa [37] reported that some trematode species (including several studied here) did not affect growth, and that some slightly stunted growth. It is possible that growth was different 25 years ago in the northern part of the California horn snail's range, where and when Sousa's work was carried out. However, it is also possible that trematodes did increase growth in Sousa's study, but that this went undetected due to methodological artifacts arising from the overdispersed growth characterizing these species. First, for overdispersed data, lower sample sizes underestimate the mean [52,53]. Therefore, in Sousa [37], the relatively low sample sizes for individual trematode species compared to uninfected snails may have yielded underestimates of growth for trematode species. Additionally, the application of standard ANCOVA to overdispersed data could have also contributed to misleading conclusions. Ongoing studies of California horn snail growth in northern populations will help to clarify these conflicting results. In another study, Lafferty [26] quantified growth for snails infected by a single trematode species (euha) and for uninfected snails. He reported that 'euha' grew slower than uninfected snails. In the current study, 'euha' grew more slowly than did uninfected females, but at a rate similar to uninfected males. Because Lafferty pooled males and females in his uninfected class, uninfected snails would have grown faster on average than did 'euha'-infected snails. Therefore, Lafferty's findings are not contradictory but are expected given the data presented here.

Instead of using dominance rank as a proxy, this study directly estimated field rates of differential extrinsic mortality based on the probability of being killed by dominant species. This turned out to be important because there was a lack of complete correspondence between dominance rank and extrinsic mortality. Two subordinate trematode species experienced the lowest levels of extrinsic mortality. One of these species (renc) suffered relatively low differential mortality because it appears to tolerate co-infection with some low- and mid-ranking species, consequently occurring relatively frequently in mixed-species infections. Species such as 'renc' appear to gain at least a partial refuge from co-infecting trematodes by using a different tissue site within the infected hosts than that used by most of the trematode species (using the mantle, versus the visceral mass) [30,54,55]. The low differential mortality for the other subordinate species (smcy) occurred because the bulk of its population recruited to areas in the estuaries where dominants did not occur, driving the lack of being killed by dominants. This is interesting, because the typical situation for guilds of trematode parasitic castrators is for spatial and temporal covariance in species' distributions to increase overall levels of competitive loss [56]. Future ecological study will examine whether certain species typically gain spatial refugia, despite this not being the overriding case in these guilds. It may also be important to conduct the research over broader spatial scales to increase the likelihood of detecting such refugia.

Multiple infections by the same trematode species can occur naturally in other larval trematode-snail systems [e.g., [57-60]]. Such multi-clone infections may have occurred undetected in this study. Theory predicts that increased frequency of multi-clone infections, and the resulting increase in intraspecific competition, can select for greater parasite reproductive effort [e.g., [61-63]]. For the species in this study, there is no data on the extent of intraspecific competition. However, one would predict that the relative importance of intraspecific competition would be greater for those species with a lower risk of mortality from dominant species. If increased intraspecific competition selects for increased reproduction at the expense of growth, it would diminish the effects observed in the current study. However, the relationships of growth and reproduction with mortality caused by interspecific antagonism were all strong (all |*r*|s between 0.81 and 0.88). Nevertheless, there was some unexplained variation, part of which might be explained by differences in the nature of the intraspecific interactions characterizing these parasitic castrators.

## Conclusions

Broadly, this study illustrates that speciose guilds of parasitic castrators (body snatchers) may allow uniquely powerful comparative tests concerning the forces promoting adaptive evolution. Previous work has shown that trematode parasitic castrators can affect the life history of their uninfected hosts [reviewed in [17]]. For example, cross-population work [64-66] has documented that increased extrinsic mortality (castration by trematodes) may select for hosts that mature at smaller sizes, a pattern first documented for the California horn snail [64]. Speciose parasitic castrator guilds readily allow us to go further than single-species studies. Many species (the castrators and their uninfected hosts) live in the same environment and use the same physiological machinery for growth and survival. However, these same species differ in important, quantifiable elements of life history. Therefore, we may gain unique insight concerning the evolution of allocation strategies by examining uninfected and parasitically castrated hosts simultaneously and relative to one another, particularly when there are several species of parasitic castrators in the assemblage [10]. The specific findings of this report support the idea that extrinsic mortality influences allocation to growth and reproduction in a way that contributes to the diversity of life histories that we see across species in nature.

## Methods

### Study system

The California horn snail, *Cerithidea californica *(Haldeman 1840) [67] (Potamididae: Prosobranchia), lives in salt-marsh dominated estuaries from central California, USA to Baja California Sur, Mexico [68,69]. The snails primarily feed on benthic diatoms [70,71]. Resource abundance limits both growth and reproduction of the California horn snail [26,72,73], further justifying the general expectation of resource allocation trade-offs.

Throughout its range in California, these snails grow and reproduce from spring through fall (March-October) and cease growth and reproduction during the winter (November-February) [37,69,74]. Maximum longevity for these snails is at least 6-10 years [24,37,74], and this appears to be the case for uninfected as well as infected snails (R.F. Hechinger and J. Lorda, unpublished analyses).

At least 18 trematode species parasitically castrate California horn snails [75,76]. A trematode infects a snail with a miracidium larva that either swims to infect the snail, or hatches after the snail ingests the trematode egg. After infection, the trematode parthenitae clonally replicate and produce free-swimming offspring (cercariae). These offspring infect second intermediate hosts (various invertebrates and fishes) where they form cysts (metacercariae). The trematodes infect bird final hosts when birds eat second intermediate hosts. The trematodes sexually produce offspring in the birds. The eggs pass with bird excreta to subsequently infect snails. Some of these and similar bird-dispersed trematodes exhibit little to no local genetic structure, being comprised of more broadly distributed, regionally panmictic populations [e.g., [77-79]].

### Mark and recapture

Approximately 20,000 snails were captured, marked, and released during mid-summer 2005 (the end of July). California horn snail performance varies at the fine scale, among sites within estuaries [26,37,69,74,80]. Therefore, to estimate the growth over a wide range of growing conditions, I conducted this study at 18 sites spread throughout three estuaries bracketing southern California: 8 sites at Carpinteria Salt Marsh (CSM) in the north, 6 sites at Los Peñasquitos Lagoon (LPL) and 4 at Tijuana Estuary (TJE) in the south. All sites were in tidal creeks, except for four sites at CSM that were in pans and "back flats" (bare mud bordered by *Salicornia virginica *salt marsh). I selected the sites for interspersion throughout each estuary and because they have all been used in previous studies [namely, [76,81-83]].

Approximately 1,000-1,300 snails were collected from less than 100 m^2 ^at each of the 18 study sites and taken to the laboratory for marking. I obtained a wide range of sizes: 8-43 mm in shell length. Each shell was processed by cleaning with a toothbrush, rinsing in fresh water, air-drying, and marking with two coats of enamel-based yellow spray-paint. This marking procedure does not appear to influence snail growth (see Additional file 1). All snails were released from 25 July - 1 August 2005.

Snails were recaptured during two time-periods, reflecting "growing-times" of 13.5 and 22 weeks. 1,277 snails were recaptured from 16 of the 18 sites in all three estuaries during winter 2005 (i.e., from the end of November through December), Assuming snails ceased growth on 1 November (see above), these snails were in the field for 92-100 growing-days (a mean of 13.5 weeks). During spring 2006 (26-27 April), an additional 642 snails were recaptured from 10 sites at LPL and TJE (including from one of the two sites from which no snails were recovered during the previous collection). Assuming growth resumed on 1 March 2006, this batch of snails grew for 153-156 days (a mean of 22 weeks). Overall, 1,919 snails were recaptured from 17 of the 18 study sites (snails were not found at one site), with a mean of 114 snails from each recapture site. This is about 10% of the total initially marked and released.

### Quantification of growth and parasitism

Several aspects of shell growth were quantified for each snail. The marking technique indicated initial size. New shell growth was evident as the paint free shell deposited beyond the marked initial shell. Following previous work [47,84,85], degrees of growth around the columnellar axis was quantified. The initial and final sizes for length and width measured to the nearest 0.1 mm. The volume of growth was the (aperture area) × (degrees of growth/360), expressed in mm^3^. To calculate aperture area, I equated it to a circle with a radius being 1/4 the snail width. I calculated initial volume by approximating shell volume to the volume of a cone. Across all measured snails, the cube root of volume linearly scaled with total length as: volume^1/3 ^= (0.326) × (length) + 0.497 (ordinary least squares regression: *R*^2 ^= 0.889, *P *< 0.00005, *n *= 1918).

Each snail's gender and trematode infection status was determined by examining dissected snails and trematode stages with a stereomicroscope. I identified all trematodes to species following an identification key created for these trematode species [75] (with minor modification—Hechinger and Huspeni, unpublished work).

### Differential extrinsic mortality

For each trematode species, I used the formulas in Lafferty et al. [33] to estimate the proportion of individuals killed by dominant species. The formulas require information on the species' dominance relationships. I used a slightly modified version of dominance hierarchies previously published for these species [29,30,79] (Additional file 1, Figure S1). The formulas simply apply the observed prevalence of each trematode species in "dominant-free" snails to the portion of the snail population infected with dominant trematode species. This number (minus any observed co-infections with dominants) provides the estimate of the number of subordinate infections killed by dominant species. Because the intensity of competition varies at the fine scale [33], I calculated differential mortality for each site at which a trematode species occurred and then took the average across all sites (weighting by observed prevalence).

For uninfected snails, to calculate differential "mortality", I simply took the average gender-specific prevalence of trematode infection across all sites (that is, the average risk of parasitic castration across all samples). I examined uninfected males and females separately, because host genders may have different susceptibilities and, consequently, different allocation patterns. Further, Sousa [37] documented that small females grew faster than small males.

### Relative reproductive allocation and 'gonadosomatic' indices

Reproductive effort is the proportional allocation of total energy to reproduction [1,4,86,87]. The gonadosomatic index (reproductive tissue mass/total mass) is commonly used to reflect reproductive effort in animals and plants [1]. Parasite reproduction is the primary function of trematode tissue in a castrated host. Therefore, the mass of pure trematode tissue in a snail divided by the total infected snail tissue mass is a gonadosomatic index for these parasitic castrators. Hechinger et al. [10] present the gonadosomatic indices (as "relative mass") for all but one of the trematode species of the current study. For the single species (renc) not included in Hechinger et al. [10], I used the gonadosomatic index of its close taxonomic and ecological relative, *Renicola buchanani*.

A gonadosomatic index has the obvious limitation that it measures standing-stock rather than actual rates of energy allocation [1]. In a comparative analysis, the gonadosomatic index could still function as useful index of reproductive effort if different species had equal rates of allocation to given masses of somatic and reproductive tissues. However, the growth differences revealed in this study indicated that species strongly differ in allocation rate to somatic tissues. Therefore, to better quantify reproductive effort, I calculated an index, "relative reproductive allocation," that captured the variation in growth allocation documented in the current study. To do this for each species, I used the gonadosomatic indices at the mean-sized snail [the summer values reported in [10]], divided by the proportional allocation to growth at the mean-sized snail. I determined the proportional allocation to growth by running a generalized linear model (GzLM) on the growth data from the current study. This GzLM was structured with the same predictor variables as the one I used to depict absolute growth (see *Analysis *and Table 1). The relative reproductive allocation index simplifies to (reproductive tissue mass)/(new growth mass). Thus, this index more accurately reflects reproductive effort than would a gonadosomatic index ignoring variation in growth. This index still suffers from using a static numerator (the mass of reproductive tissues), but there is limited cross-species evidence that the mass of trematode tissue in a snail is directly proportional to the mass of offspring produced (see the analyses of McCarthy et al.'s [44] data in Hechinger et al.[10]).

Based on gonad size, the gonadosomatic index for uninfected females (7.7%) is lower than that for uninfected males (14.6%) [10]. However, as Hechinger et al. [10] pointed out, unlike males, females have other substantial reproductive tissues, including a large pallial gonoduct and an ovipositor [88]. To provide a more accurate index of female reproductive allocation, I determined a female gonadosomatic index that included the mass of these additional tissues. I obtained fresh weights for the pallial gonoduct, ovipositor, and total soft tissue for 20 uninfected females. The relative mass of these reproductive tissues was 7.2 ± 0.5% (± S.E., standard error). Adding this to the reported 7.7 ± 0.8% relative mass for ovaries yields the more accurate female gonadosomatic index of 14.9 ± 0.9% used in this study.

### Species differences genetic or plastic?

I sought to determine whether species' differences in growth rates represent consistent relative differences or general plastic responses to varying local risk of being killed by trematodes. If individuals did plastically respond to local risk, we would predict that, within a species, individuals would decrease allocation to growth at sites with higher extrinsic mortality. To test this idea, I asked whether individual growth rates within each species correlated across sample sites with differential mortality risk. Differential mortality risk for each trematode species at a site was the local prevalence of dominant trematodes. For uninfected snails, it was the overall trematode prevalence (that is, the overall risk of castration).

### Analysis

Because snail growth was overdispersed, with many individuals growing little or not at all, and some growing quite a bit (Additional file 1, Figure S2), I analyzed individual snail absolute and proportional growth using generalized linear models (GzLMs) with a log-link and a Poisson error distribution with an overdispersion parameter [89,90]. Table 1 lists the predictor variables. Because I was solely interested in controlling for site variation, I incorporated site identity as a fixed factor. Because snails from CSM came only from the first growing period (summer to fall), I also nested the effect of growing-time within estuary. One advantage of using multiple species in a single model is that the increased sample size allows better estimation of, and control for, site growing conditions and growing-time differences. I ensured that the significance of the "species" effect was not solely due to differences between infected and uninfected snails. To do this, in addition to examining the statistical significance of each species' parameter estimate in the GzLM, I performed all 45 of the species' pair-wise comparisons (contrasts). I assessed contrast significance at the nominal *P *= 0.05 level, and also when holding the family-wide False Discovery Rate [91] to 0.05. Unless otherwise indicated, all significance tests from GzLMs used likelihood-ratio *X*^2 ^statistics (and some of the contrasts used Wald tests, when likelihood-ratio tests were unavailable).

For correlation significance tests, I used randomization tests [92,93] with 100,000 iterations each. For other analyses, I employed standard parametric tests, including general linear models (GLMs, [94]).

For parametric tests, I ensured assumptions were met regarding distribution of the error terms by inspecting residual versus predicted plots and normal quantile plots with Lillifors curves for GLMs, and Studentized deviance versus predicted plots for GzLMs [89,94]. All *P*-values are two-tailed. I used the software JMP ver. 7.0.2 for parametric tests, and Resampling Stats for Excel ver. 3 for randomization tests.

**Figure 4:**
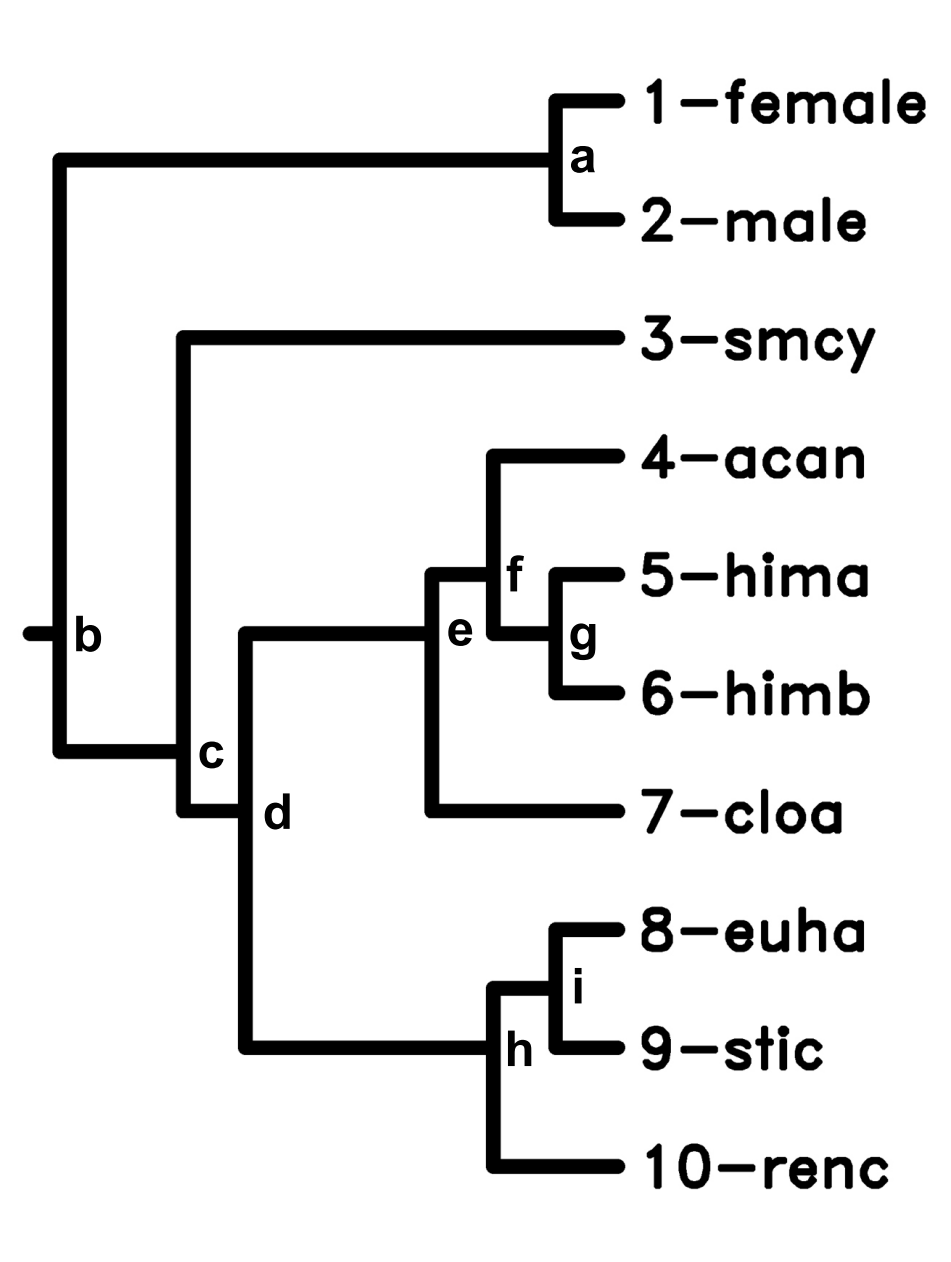
**The phylogenetic tree, used to generate independent contrasts, representing the relationships among the trematode parasitic castrator species and uninfected snail hosts**. Trematode species codes and names are in Figure 1 and Additional file, Table S1. "Female" and "male" are uninfected California horn snails, *Cerithidea californica*. Letters represent the position of contrasts indicated in Figures 3B, 4B, and 4D. Tree composed following Hechinger et al. [10], using information from Olsen et al. [97] and Yamaguti [98].

To ensure independence of data and to alleviate potential confounds due to common ancestry, I also performed analyses using phylogenetically independent contrasts [95,96]. Following Hechinger et al. [10], I determined the topology of the phylogenetic tree characterizing the study species (Figure 4) using the phylogeny of trematode families provided by Olsen et al. [97] and information on generic or subfamilial relationships [98] to resolve within-family topography. Because male and female uninfected snails grew differently, I included the separate genders in the tree. Although the different genders clearly do not represent independent evolutionary lineages, this allowed examination of how the contrasts between uninfected snail genders compared to the contrasts between species. I used branch lengths of one, because we do not know how evolution occurs along these lineages. The software, COMPARE 4.6b (E.P. Martin, 2004, Indiana University), generated the contrasts. The contrasts ensure independence of the data points, and I used randomization correlation tests to obviate excessive concern about potential heteroscedasticity. Regression through the origin complemented the correlations to confirm contrast relationships.

Of the 1,919 recaptured snails, I excluded from growth estimates 60 mixed-species infections, 1 outlier (Additional file 1, Figure S2), and 92 snails with undetermined initial size, gender, or infection status (including immature infections). Excluding mixed-species and immature infections removes ensures that most recaptured infections represent the original infection status. If some rare infections completely changed status during the course of this investigation, they would tend to obscure patterns, not create them. Also, because lower sample sizes give underestimates of means for overdispersed data [52,53], I additionally excluded 80 infections by nine rare trematode species (with *n *between 2 and 16). For the eight trematode species with over 20 individuals sampled (Figure 1; Additional file 1, Table S1), there was no evidence that estimates of growth rates increased with sample size for either total growth allocation (Additional file 1, Figure S4A, *r *= -0.03, *P *= 0.95, *n *= 8) or growth at the mean-sized snail (Additional file 1, Figure S4B, *r *= 0.18, *P *= 0.67, *n *= 8). Thus, data from 1,686 snails (1,043 uninfected snails and 643 infected by one of eight trematode species) went into the main growth analysis (Table 1) and into the derivative data. Julio Lorda and I plan to present, elsewhere, analyses of the ecological relevance of these data.

## Authors' contributions

RFH conceived the study, acquired the data (with assistance of people in the acknowledgments), analyzed the data, and wrote the paper.

## Author information

RFH is one of the lead investigators of the Ecological and Evolutionary Parasitology research group at the University of California, Santa Barbara. Two major intellectual goals underlie much of his research. One goal is to evaluate the importance of parasites and parasitism to various ecological patterns and processes. The other goal is to reap the benefits of using parasites and their hosts to tackle general ecological and evolutionary theory. RFH is inordinately fond of trematodes, parasitic castration, and coastal wetlands.

## Supplementary Material

Additional file 1 **Appendix**. PDF file with supplementary experiments, analyses, tables and figures. Click here for file
