# Supplementary material for: Mortality affects adaptive allocation to growth and reproduction: field evidence from a guild of body snatchers
Source: BMC Evol Biol. 2010 May 7;10:136. doi: 10.1186/1471-2148-10-136 (PMC2887408; doi:10.1186/1471-2148-10-136)
Supplement: Additional file 1 — Appendix. PDF file with supplementary experiments, analyses, tables and figures. [file 1471-2148-10-136-S1.PDF]

Appendix

Supplementary experiments, analyses, tables and figures

Contents

- **Marking technique did not influence growth**
- **Snail gender influences growth primarily for uninfected snails**
- **Trematode and gender effects are consistent among estuaries**
- **Trematodes do not disproportionally infect faster growing snails**
- **Reproductive allocation did not spuriously correlate with differential mortality**
- **Table S1 - Taxonomic family, name, and coding for the focal trematode species of this study.**
- **Table S2 - Sample sizes by estuary and gender for the species included in the initial assessment of whether gender effects varied with infection status.**
- **Figure S1 - Dominance hierarchy for the trematode parasitic castrators of California horn snails.**
- **Figure S2 - Growth versus initial size for 1,687 individual California horn snails.**
- **Figure S3 - Gender effects on growth are diminished for infected snails.**
- **Figure S4 - Assessment of sample size bias for estimates of growth.**
- **Figure S5 - Trematode infection does not alter skewness of uninfected snail growth distribution.**
- **Figure S6 - Mean growth rates of trematode parasitic castrator infected snails and uninfected snails do not appear to respond plastically to the local risk of being killed by dominant species of trematodes (local risk of castration for uninfected snails)**
- **Appendix References**

Marking technique did not influence growth

I performed a field experiment to determine whether marked snails grew differently than did infected snails. At the same time as the rest of the marking and releasing, I collected just over 200 snails from each of two additional channel sites at CSM. I randomly split the snails from each site into two groups. One-half was painted using the marking technique described above. Field assistants placed the marked and unmarked snails in a single mesh cage (1 m diameter) at each original collection site. After 3.5 months of growth, I retrieved the marked and unmarked snails from one site (the cage collapsed at the other of the two sites, releasing all the snails). In the lab, I determined whether the snails were alive or dead and quantified their shell length.

Marking did not appear to affect snail growth or mortality. The initial mean size of the marked group was statistically indistinguishable from the unmarked group (24.9 ± 4.4 mm (SD) vs. 25.3 ± 4.5 mm (SD); two-sample randomization test (Edgington 1995), $P = 0.62$, $n = 211$). I recovered 97 of the 106 painted snails (92%) and 79 of the 105 unmarked snails (75%). The size-frequency distributions largely overlapped, and painted and unpainted snails did not differ in mean length among alive (26.2 ± 0.29 mm (SEM) vs. 26.8 ± 0.44 mm; two-sample *t*-test, $t_{138} = 0.96$, $P = 0.34$) or dead snails (25.2 ± 0.67 mm vs. 25.1 ± 0.67 mm; $t_{138} = 0.14$, $P = 0.88$). Finally, painted and unpainted snails had statistically indistinguishable mortality (19% vs. 23%) (likelihood ratio X^2 test; $X^2 = 0.48$, $P = 0.49$, $n = 176$).

Snail gender influences growth primarily for uninfected snails

I used several approaches to examine whether gender effects varied with infection status. Because estimates of means are biased downward at low sample sizes for overdispersed data (Pacala and Dobson 1988, Gregory and Woolhouse 1993), I first examined gender effects on a subset of the data. This data subset included uninfected snails and the four most common trematode species in estuaries where sample size exceeded 65 (Figure 1; Additional file 1, Table S2). Sex ratios were also quite even (Additional file 1, Table S2). I tested the effect separately for each species by nesting gender (and its interaction with initial size) within species, including uninfected snails as a species. There were no significant main effects of gender within species (all $P > 0.44$) and gender interacted significantly with initial size only for uninfected snails ($X^2 = 7.5$, $P = 0.0063$; all other $P > 0.15$). However, all the parameter estimates for trematodes interactions between gender and size consistently were in the same direction as that for uninfecteds. This suggested that there might be a lack of power to detect potentially weaker gender effects for infected snails. I therefore ran a model pooling these trematode species, nesting gender within infected or uninfected. Here, gender did interact with size to influence growth within both infected and uninfected snails (respectively, $X^2 = 9.9$, $P = 0.0017$ and $X^2 = 8.2$, $P = 0.0041$). The same results occurred when I included all species with $n > 20$ ($X^2 = 6.2$, $P = 0.013$ and $X^2 = 6.8$, $P = 0.0090$). Thus, gender differences are undetectable or far smaller for infected snails than for uninfected snails, and appear to disappear rapidly with an increase in size (Additional file 1, Figure S3). Given the large differences between uninfected male and female snails, I kept them separate in the main analyses.

Trematode and gender effects are consistent among estuaries

I examined whether infection and gender effects on growth were consistent across estuaries. Using a GzLM, I separately tested effects for each estuary by allowing estuary to interact with infection status and with gender (nesting the estuary x gender interaction within infection status; effectively, a three-way interaction). I first ran an analysis pooling the 644 individuals of the eight most common trematodes to assess the consistency of gender effects on growth for uninfected and infected snails. As in the main model with species, growth rates did not vary among estuaries (X^2 = 0.53, P = 0.77, n = 1658) There was no detectable difference in how infection affected growth across estuaries (interaction term, X^2 = 1.56, P = 0.46, n = 1658). Infected snails grew faster than did uninfected snails in each estuary: the non-significantly different maximum likelihood estimates being 1.26 (1.12-1.42, 95% CI) times faster at CSM; 1.35 (1.18-1.54) times faster at LPL, and 1.44 (1.21-1.71) times faster at TJE. Further supporting consistent effects across wetlands, a series of contrasts comparing the infection status x estuary interaction between all estuary pairs detected no significant differences (Wald tests, all $P > 0.23$). Additionally, there was no sign that gender affected growth differently in different estuaries for both infected and uninfected snails (interaction term X^2 = 1.86, P = 0.76, n = 1,658). Here, gender did not significantly affect growth ($P > 0.50$) nor interact with initial size ($P > 0.19$). However, the interaction with size for uninfected females was consistently larger for uninfected than for infected females (congruent with the indication of weaker gender effects documented above). Additionally, the maximum likelihood parameter estimates for infected and uninfected females within each estuary were consistent in direction; further indicating effects are consistent across wetlands. These results were mirrored in parallel analyses including all trematodes with $n > 10$ (n = 1,711) and all identified trematodes (n = 1,737). Thus, overall and within each estuary, small females grow faster than do males, particularly so for uninfected snails.

The consistency among wetlands concerning trematode and gender effects bolsters performing a single-analysis examining the average effect of each trematode species across all three estuaries. Additionally, consistent effects for the trematode species should perhaps be expected, given that these and similar bird dispersed trematodes exhibit little or no local genetic structure, being comprised of more broadly distributed, panmictic populations (e.g., Huspeni 2000, Miura et al. 2005, Keeney et al. 2009).

Trematodes do not disproportionally infect faster growing snails

The increased growth rates for trematodes (operating stolen host bodies) could potentially arise by trematodes disproportionately infecting faster growing snails. Evidence from the data indicates this is not the case. If trematodes disproportionally infected faster growing snails, increasing prevalence of infection would increasingly alter the distribution of growth observed for uninfected snails. Specifically, increasing infection prevalence would make the distribution of uninfected growth less skewed. This would occur as infection removes a greater proportion of uninfected snails from the tail of the distribution that includes faster growers. However, skewness in growth did not associate with prevalence across study sites (Additional file 1, Figure S5; regression: R^2 = 0.0055, P = 0.79), including after correcting for the potential covariate of sample size (GLM: R^2 = 0.0004, $F_{1,14}$ = 0.0048, P = 0.95). Thus, trematodes appear not to disproportionally infect faster-growing snails. This evidence combines with the knowledge that

trematode species differently alter other components of the stolen snail bodies' energy allocation (i.e., the gonadosomatic index (Hechinger et al. 2008)) to indicate that the trematode castrators also differentially allocate resources to growth of the stolen host bodies.

Reproductive allocation did not spuriously correlate with differential mortality

I made sure that the strong negative relationship between growth and mortality did not artifactually force a positive correlation between reproductive allocation and mortality. To do this, I performed a two-step randomization test. First, I randomly matched species' gonadosomatic indices and growth allocation values and, from these, calculated relative reproductive allocation. The second stage was a standard randomization correlation test (see below) between these relative reproductive allocation values and differential mortality. This two-stage process was iterated 100,000 times to generate the null distribution of correlation coefficients. Therefore, this test incorporates into the null distribution any spurious association between the variables, instead of assuming the null correlation is zero (see Jackson and Somers 1991). However, on the null hypothesis, the correlation coefficient for relative reproductive allocation and mortality was centered on zero. This and the resulting low *P*-value demonstrate that the observed positive relationship was not artifactually positive.

Table S1 - Taxonomic family, name, and coding for the focal trematode species of this study.

Family	Species	Species letter code	Species number code
Cyathocotylidae	small cyathocotylid[a]	smcy	3
Echinostomatidae	*Acanthoparyphium spinulosum*	acan	4
	Himasthla rhigedana	hima	5
	Himasthla sp. B.[a]	himb	6
Philophthalmidae	*Cloacitrema michaginensis*	cloa	7
Heterophyidae	*Euhaplorchis californiensis*	euha	8
	Stictodora hancocki	stic	9
Renicolidae	*Renicola cerithidicola*	renc	10

[a]Recognized as species, despite not having been formally described.

Table S2 - Sample sizes by estuary and gender for the species included in the initial assessment of whether gender effects varied with infection status.

Species code[a]	Estuary	f	m	Total
smcy	LPL	66	75	141
hima	CSM	33	33	66
himb	CSM	42	74	116
euha	CSM	51	45	96
u	CSM	113	143	256
u	LPL	283	204	487
u	TJE	140	160	300
			total	1462

[a]Codes as in Figure 2 and Additional file 1, Table S1, “u” stands for uninfected snails.

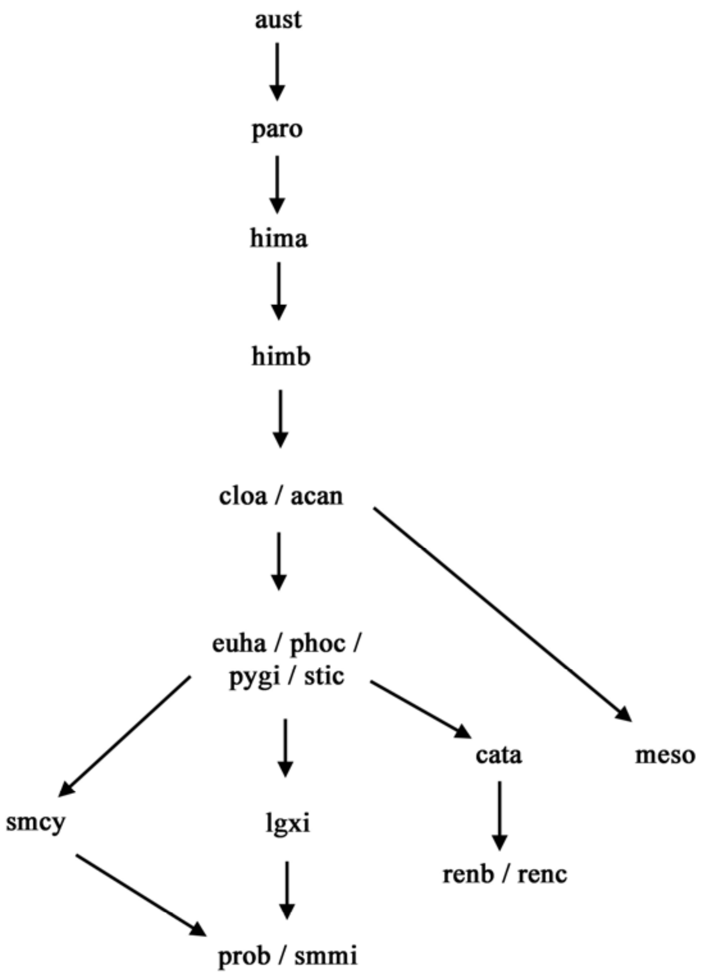

Figure S1 - Dominance hierarchy for the trematode parasitic castrators of California horn snails. Information on dominance relationships from Kuris (1990), Sousa (1993), and Huspeni (2000). Species codes in Figure 1 and Additional file 1, Table S1. Arrows indicate transitive dominance relationships (e.g., a species dominant to species A kills all species killed by species A). Species at the same level are treated as co-dominant, each winning 50% of time when in co-infections (see Lafferty et al. 1994). Two species, ‘smmi’ and ‘stic’, were not recognized in previous (Kuris 1990, Sousa 1993, Huspeni 2000) hierarchies.

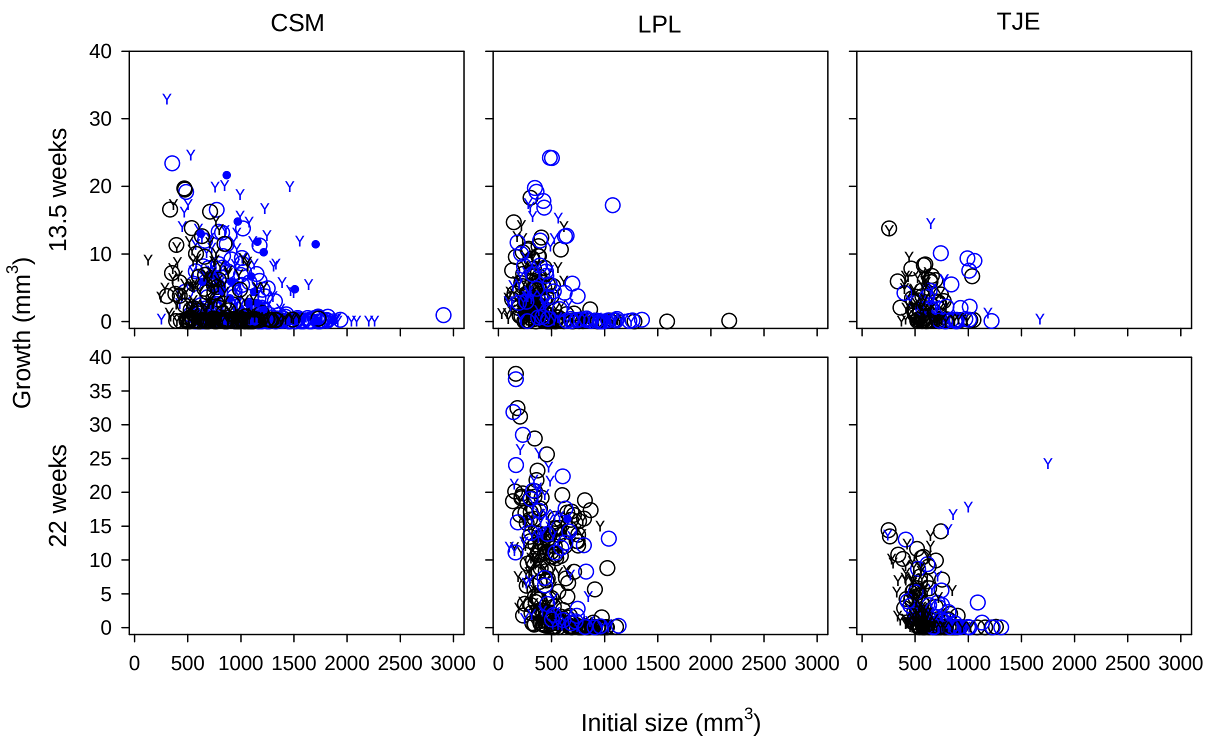

Figure S2 - Growth versus initial size for 1,687 individual *Cerithidea californica* snails (1,043 uninfected snails and 644 infected by one of eight trematode parasitic castrator species) in three southern California estuaries. CSM = Carpinteria Salt Marsh, LPL = Los Peñasquitos Lagoon, and TJE = Tijuana Estuary. Circles represent females; Ys, males; black, uninfected snails; blue, infected snails; the small filled circles represent 28 infected individuals with undetermined host gender. The infected male outlier in the bottom right panel was excluded from analyses.

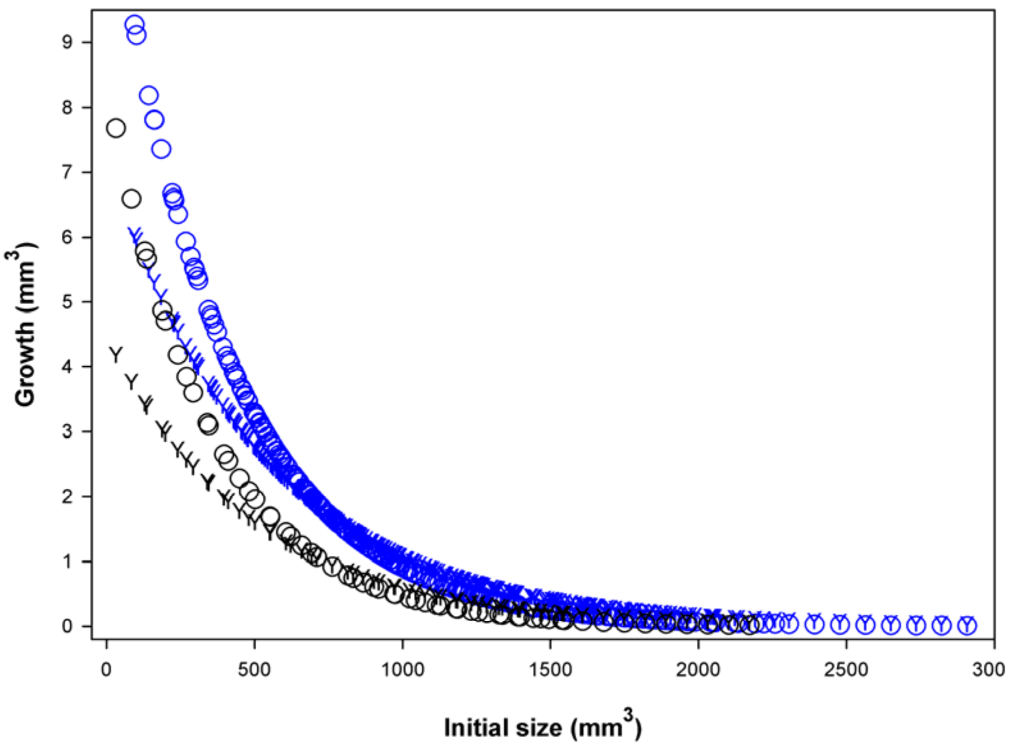

Figure S3 - Gender effects on growth are diminished for infected snails, being detectable only when pooling individuals across species. Individual growth is size-specific mean growth, using a GzLM to control for the growing conditions at a site, growing time, and to standardize data to reflect 3.5 months of growth in Carpinteria Salt Marsh channels. Circles represent females; Ys, males; black, uninfected snails; and blue, infected snails.

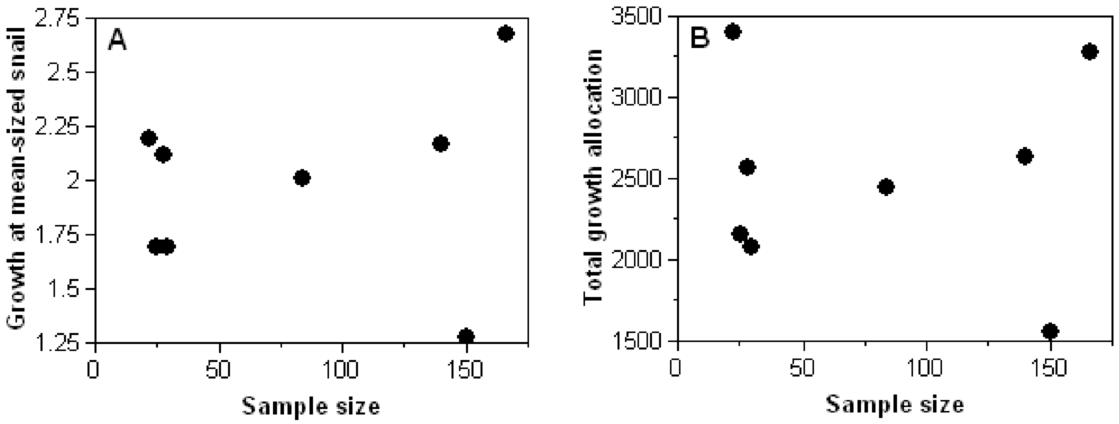

Figure S4 - Assessment of sample size bias for estimates of (A) total growth allocation (area under the growth curves in Figure 2) and (B) growth rates at the mean-sized snail. The lack of a positive trend with increasing sample size indicates that there was no systematic underestimate of growth rate.

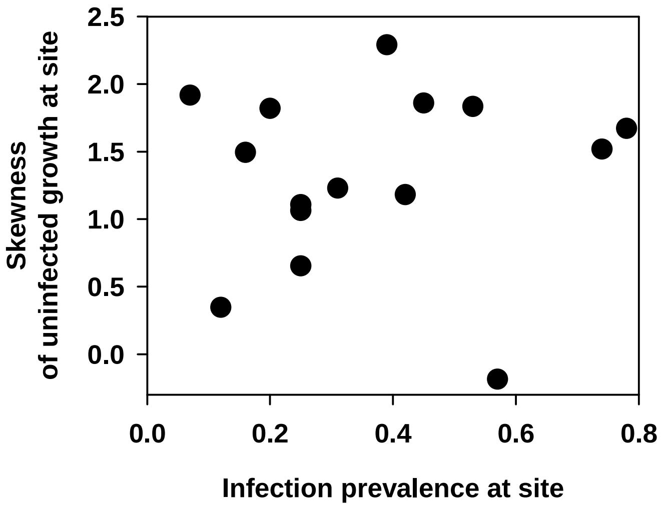

Figure S5 - Trematode infection does not alter skewness of uninfected snail growth distribution. This provides evidence that trematodes do not disproportionally infect faster growing snails. Skewness calculated on growth residuals from a generalized linear model controlling for covariates (Table 1).

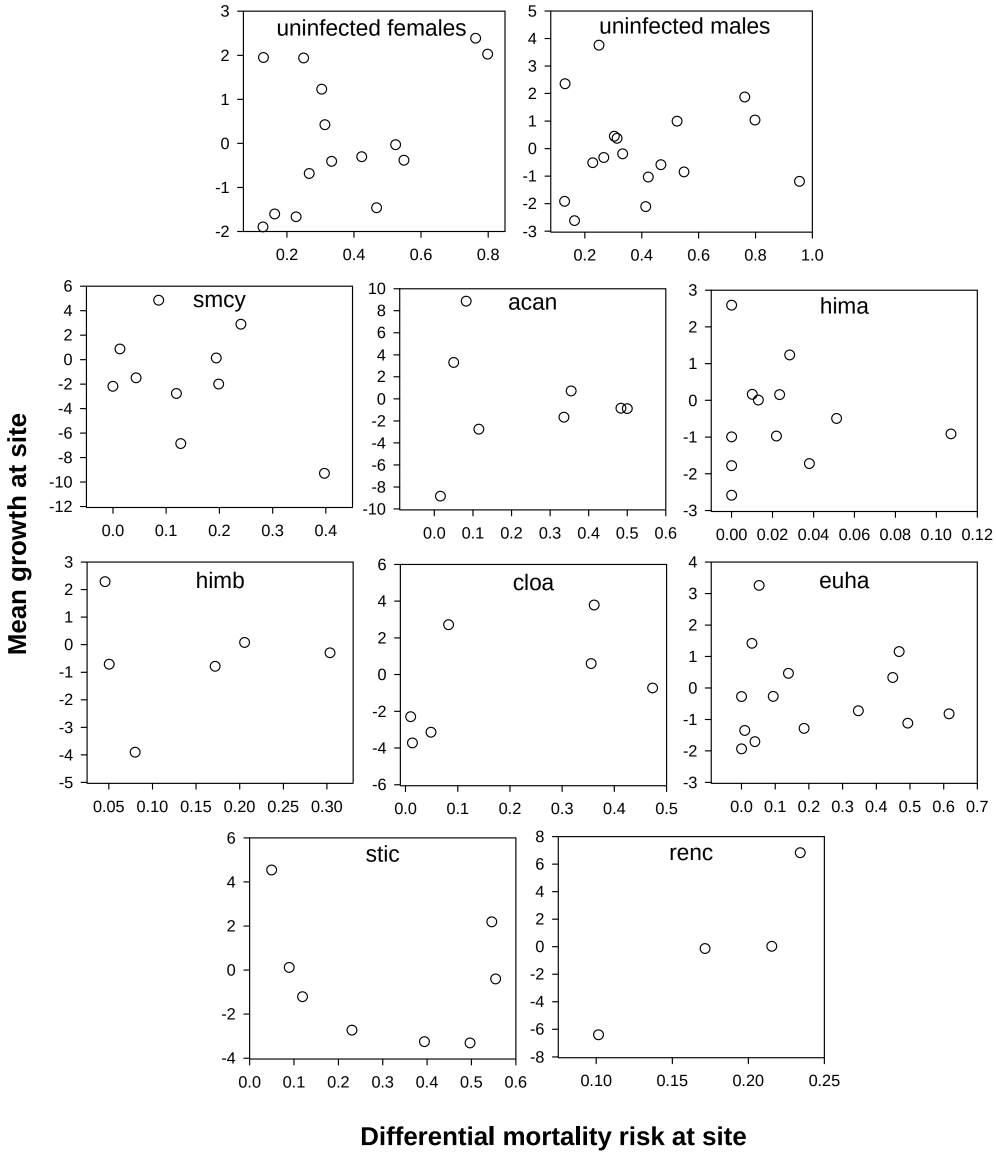

Figure S6 - Mean growth rates of trematode parasitic castrator infected snails and uninfected snails do not appear to respond plastically to the local risk of being killed by dominant species of trematodes (local risk of castration for uninfected snails). Panels show site-specific data for each trematode species and uninfected male and female snails. Values for growth are the deviations from a generalized linear model (GzLM) controlling for estuary, habitat, growing time, snail size, and species. I tested the relationship using a general linear model (GLM) nesting risk within species, and weighting sites by the number of individuals sampled. A GLM using risk, species, and their interaction term gave almost identical results, including after dropping the non-significant interaction (P = 0.49). Almost identical results also occurred when species was removed from the model ($F_{1,100}$ = 1.86, P = 0.18).

Appendix References

Edgington, E. S. 1995. Randomization tests. 3rd edition. Marcel Dekker, Inc., New York.

Gregory, R. D. and M. E. J. Woolhouse. 1993. Quantification of parasite aggregation: a simulation study. Acta Tropica **54**:131-139.

Hechinger, R. F., K. D. Lafferty, F. T. Mancini III, R. R. Warner, and A. M. Kuris. 2008. How large is the hand in the puppet? Ecological and evolutionary effects on body mass of 15 trematode parasitic castrators in their snail host. Evolutionary Ecology **DOI 10.1007/s10682-008-9262-4**.

Huspeni, T. C. 2000. A molecular genetic analysis of host specificity, continental geography, and recruitment dynamics of a larval trematode in a salt marsh snail. PhD. University of California, Santa Barbara, Santa Barbara.

Jackson, D. A. and K. M. Somers. 1991. The spectre of 'spurious' correlations. Oecologia **86**:147-151.

Keeney, D. B., T. M. King, D. L. Rowe, and R. Poulin. 2009. Contrasting mtDNA diversity and population structure in a direct-developing marine gastropod and its trematode parasites. Molecular Ecology **18**:4591-4603.

Kuris, A. M. 1990. Guild structure of larval trematodes in molluscan hosts: prevalence, dominance and significance of competition. Pages 69-100 *in* G. W. Esch, A. O. Bush, and J. M. Aho, editors. Parasite communities: patterns and processes. Chapman and Hall, London.

Lafferty, K. D., D. T. Sammond, and A. M. Kuris. 1994. Analysis of larval trematode communities. Ecology **75**:2275-2285.

Miura, O., A. M. Kuris, M. E. Torchin, R. F. Hechinger, E. J. Dunham, and S. Chiba. 2005. Molecular-genetic analyses reveal cryptic species of trematodes in the intertidal gastropod, *Batillaria cumingi* (Crosse). International Journal for Parasitology **35**:793-801.

Pacala, S. W. and A. P. Dobson. 1988. The relation between the number of parasites/host and host age: population-dynamic causes and maximum-likelihood estimation. Parasitology **96**:197-210.

Sousa, W. P. 1993. Interspecific antagonism and species coexistence in a diverse guild of larval trematode parasites. Ecological Monographs **63**:103-128.
